# Use of NHANES Data to Link Chemical Exposures to Chronic Diseases: A Cautionary Tale

**DOI:** 10.1371/journal.pone.0051086

**Published:** 2012-12-05

**Authors:** Judy S. LaKind, Michael Goodman, Daniel Q. Naiman

**Affiliations:** 1 LaKind Associates, LLC, Catonsville, Maryland, United States of America; 2 Department of Epidemiology and Public Health, University of Maryland School of Medicine, Baltimore, Maryland, United States of America; 3 Department of Pediatrics, Pennsylvania State University College of Medicine, Hershey, Pennsylvania, United States of America; 4 Department of Epidemiology, Emory University, School of Public Health, Atlanta, Georgia, United States of America; 5 Department of Applied Mathematics and Statistics, The Johns Hopkins University, Baltimore, Maryland, United States of America; Lovelace Respiratory Research Institute, United States of America

## Abstract

**Background:**

The National Health and Nutrition Examination Survey (NHANES) is one example of cross-sectional datasets that have been used to draw causal inferences regarding environmental chemical exposures and adverse health outcomes. Our objectives were to analyze four NHANES datasets using consistent *a priori* selected methods to address the following questions: Is there a consistent association between urinary bisphenol A (BPA) measures and diabetes, coronary heart disease (CHD), and/or heart attack across surveys? Is NHANES an appropriate dataset for investigating associations between chemicals with short physiologic half-lives such as BPA and chronic diseases with multi-factorial etiologies? Data on urinary BPA and health outcomes from 2003–2004, 2005–2006, 2007–2008, and 2009–2010 were available.

**Methodology and Findings:**

Regression models were adjusted for creatinine, age, gender, race/ethnicity, education, income, smoking, heavy drinking, BMI, waist circumference, calorie intake, family history of heart attack, hypertension, sedentary time, and total cholesterol. Urinary BPA was not significantly associated with adverse health outcomes for any of the NHANES surveys, with ORs (95% CIs) ranging from 0.996 (0.951–1.04) to 1.03 (0.978–1.09) for CHD, 0.987 (0.941–1.04) to 1.04 (0.996–1.09) for heart attack, and 0.957 (0.899–1.02) to 1.01 (0.980–1.05) for diabetes.

**Conclusions:**

Using scientifically and clinically supportable exclusion criteria and outcome definitions, we consistently found no associations between urinary BPA and heart disease or diabetes. These results do not support associations and causal inferences reported in previous studies that used different criteria and definitions. We are not drawing conclusions regarding whether BPA is a risk factor for these diseases. We are stating the opposite–that using cross-sectional datasets like NHANES to draw such conclusions about short-lived environmental chemicals and chronic complex diseases is inappropriate. We need to expend resources on appropriately designed epidemiologic studies and toxicological explorations to understand whether these types of chemicals play a causal role in chronic diseases.

## Introduction

The Centers for Disease Control and Prevention’s (CDC) National Biomonitoring Program – part of the National Health and Nutrition Examination Survey (NHANES) - measures over 450 chemicals in people in the US. The scientific literature is replete with publications reporting associations between US population levels of chemicals in blood and/or urine and a health outcome or biochemical indicator using NHANES data [1], [2], [3], [4], [5], [6], [7], [8]. Bisphenol A (BPA), a chemical primarily used to manufacture polycarbonate plastic and epoxy resins, has been the subject of extensive research and media attention and is one of the chemicals for which NHANES data have been used to examine such associations [9], [10], [11], [12], [13], [14], [15], [16].

Three studies [9], [10], [11] evaluated associations of urinary BPA concentrations with diabetes and cardiovascular disease using data from three different individual NHANES timeframes (2003–2004, 2005–2006, or 2007–2008). While the first of these studies [9] reported significant positive associations between urinary BPA and heart attack, coronary heart disease (CHD), angina, and diabetes for the 2003–2004 survey, the other studies which used data from the next two time intervals yielded inconsistent results.

These studies varied in their methods with respect to subject inclusion criteria, case definitions and modeling strategies. Utilization of different methodologies is not inherently inappropriate; however, even in the absence of consistent methods, a robust association should yield consistent findings.

In this paper, we analyze data from four NHANES surveys (2003–2004, 2005–2006, 2007–2008, and 2009–2010) with consistent *a priori* chosen methods to 1) reassess the evidence for associations between urinary BPA and health outcomes, 2) determine whether use of a consistent scientifically and clinically supportable methodology yields coherent results across datasets, and 3) compare our methodology and results with previous studies that examined individual NHANES surveys. Most importantly, we address a larger question: Is NHANES an appropriate source of data for investigating associations between chemicals with short physiologic half-lives such as BPA and chronic diseases with multi-factorial etiologies such as diabetes or heart disease?

## Methods

### Urinary BPA Measurements

The CDC National Center for Health Statistics data files for NHANES are available at http://www.cdc.gov/nchs/nhanes.htm. Urinary BPA data are from a subsample (Laboratory 24) of the total NHANES population. Total BPA, after hydrolysis of conjugated metabolites, was measured in urine (analyte variable URXBPH, ng/ml). The method limit of detection (LOD) is given as 0.36 ng/ml for the 2003–2004 survey and 0.4 ng/ml for the other three surveys. CDC assigns a value of the LOD/√2 for measures below the detection limit [17].

### Dependent Variables

Outcomes of interest are CHD, heart attack, and diabetes because these were the focus of several previous studies. For CHD and heart attack, we use physician diagnosis to define cases (variables MCQ160C for CHD and MCQ160E for heart attack). Information on these outcomes was available in all four surveys for participants ≥20 years of age.

Participants were categorized as having type 2 diabetes if they met at least one of the following criteria [18]:

1. physician-diagnosed diabetes (variable DIQ010; available for ages ≥1 year), or
2. fasting glucose >126 mg/dl (variable LBXGLU; available for ages ≥12 years), or
3. two-hour glucose tolerance test >200 mg/dl (variable LBXGLT; available for ages ≥12 years [test not conducted in NHANES 2003–2004]).

To limit the health outcome to type 2 diabetes, we excluded participants who started insulin at the time of diagnosis (if current age in years (variable RIDAGEMN divided by 12) minus age of diagnosis (DID040Q) was ≤ the number of years of reported insulin use (variable DID060Q)).

### Data Analysis

All multivariable analyses were controlled for *a priori* selected potential confounders including, but not limited to, those used in the previous studies [9], [10], [11]. The models included the following covariates: creatinine, age, gender, race/ethnicity, education, income, smoking, body mass index (BMI), waist circumference (WC), heavy drinking, family history of diabetes (in the analyses of diabetes) or heart attack/angina (in the analyses of CHD and heart attack), hypertension, sedentary activity, blood cholesterol, and daily energy intake. These variables are considered candidate confounders because they represent known risk factors for the health outcomes of interest [19], [20], [21]. Unadjusted BPA concentrations were included in the analysis with urinary creatinine added as a separate independent variable [22]. Covariate descriptions, sources of data that provide the rationale for considering these candidate confounders, and NHANES survey year availability are given in Table 1.

**Table 1:** Description of covariates used in the fully adjusted model.

Covariate	NHANES variable,units	Source of evidence tosupport inclusion	Categorizationmethod	Age available(yrs)
Creatinine	URXUCR, mg/dl	[22]	Continuous variable	≤6
Age^a^	RIDAGEMN, months	[9], [10], [11]	Continuous variable	0–850–80
Gender	RIAGENDR	[9], [10], [11]	Male or female	All
Race/ethnicity	RIDRETH1	[9], [10], [11]	Non-Hispanic whiteNon-Hispanic blackMexican AmericanHispanic, other	All
Education	DMDEDUC3DMDEDUC2	[9], [10], [11]	< high school, high schooldiploma [including GeneralEducational Development],and>high school/all	≤6
Income	INDFMINC, INDFMIN2	[9], [10], [11]	0-$19999$20000-$22999$45000-$74999>$74999	All
Smoking^b^	SMQ020SMQ040	[9], [10], [11]	Ever, former, current, never	≤20
BMI	BMXBMI, kg/m^2^	[9], [10], [11]	Continuous variable	≤2
Waist circumference (WC)	BMXWAIST (cm)	[9], [10], [11]	Continuous variable	≤2
Heavy drinking	ALQ150	[23], [24]	Ever drank 5 or more drinksof any kind of alcoholic beveragealmost every day (Yes/No)	≤20
Family history: diabetes	2003–04:MCQ260AA, MCQ260AB,MCQ260AG, MCQ260AH2005–06, 2007–08, 2009–10:MCQ300C	[25]	Any first degree relative (Yes/No)	≤20
Family history: heartattack/angina^c^	2003–04:MCQ260GA, MCQ260GB,MCQ260GH, MCQ260GG2005–06, 2007–08, 2009–10:MCQ300A	[25]	Any first degree relative (Yes/No)	≤20
Hypertension	BPQ020	[26], [27], [28]	Hypertensive if physician-diagnosed (Yes/No)	≤16
Sedentary activity^d^	PAD590, hr/dayPAD560, hr/day	[29], [30], [31]	Continuous variableContinuous variable	≤2
Cholesterol	LBXTC, mg/dl	[32]		3 or 6 and older
Energy intake^e^	DR1TKCAL, kcalDR2TKCAL, kcal	[33], [34]	Continuous variable: averageof days 1 and 2	All

a For the 2003–2004 and 2005–2006 NHANES surveys, data are given for ages 0 to 85 years; all participants who were age 85 or older were assigned an age of 85 to protect their confidentiality [35]. For 2007–2008 and 2009–2010, age data range from 0 to 80 years; for individuals older than 80 years of age, CDC assigned a value of 80 years.

b Former smokers – those who answered yes to variable SMQ020 (Have you smoked at least 100 cigarettes in your entire life? AND no to variable SMQ040 (Do you now smoke cigarettes); Current smokers – those who answered yes to variable SMQ040; and Never smokers – those who answered no to SMQ020 and SMQ040.

c NHANES 2003–2004 survey asked only about heart attack but at any age.

d defined as time sitting and watching TV or videos in the past 30 days and time using the computer or playing computer games over the past 30 days; excluded as a covariate for the 2007–2008 and 2009–2010 surveys as inclusion led to unacceptably small sample size (almost all sedentary data were missing for people with urinary BPA measurements).

e not available for NHANES 2009–2010.

Analyses of the association between urinary BPA and each health outcome were conducted separately for each of the four NHANES surveys. All analyses used multivariable logistic regression models with results expressed as adjusted odds ratios (ORs) with corresponding 95% confidence intervals (CI) and P-values. The ORs for continuous variables in these models, including urinary BPA, reflect the change in odds of outcome per unit change of exposure.

In addition to assessing the results across survey years, we conducted pooled analyses of all four NHANES datasets. To assess the impact of covariates on study results, the pooled analyses used six models, each involving progressively more covariates. The baseline model (Model 0) included only BPA and survey year. Model 1 also controlled for creatinine, age, gender, ethnicity, education and income. Model 2 used previously included covariates plus smoking and drinking. Model 3 added BMI and waist circumference and Model 4 further added hypertension and total cholesterol. The final model (Model 5) included all of the above plus family history, and thus controlled for all *a priori* selected covariates.

CDC’s weighting factors were incorporated in the analysis. Missing data were handled by including only those individuals for whom all covariates were available. Analyses were carried out using SAS 9.3 statistical software (SAS Institute, Cary, NC).

## Results

### Our Findings across All Surveys

Tables 2, 3 and 4 show the results of the fully adjusted model for associations between urinary BPA and CHD, heart attack, and diabetes, respectively, for the four NHANES surveys. Urinary BPA was not significantly associated with any of the adverse health outcomes for any of the NHANES surveys with ORs (95% CI) ranging from 0.996 (0.951–1.04) to 1.03 (0.978–1.09) for CHD, from 0.987 (0.941–1.04) to 1.04 (0.996–1.09) for heart attack, and from 0.957 (0.899–1.02) to 1.01 (0.980–1.05) for diabetes.

**Table 2:** Association between BPA and CHD using fully adjusted model (creatinine, age, gender, ethnicity, education, income, smoking, heavy drinking, BMI, waist circumference, energy intake, family history of heart attack, hypertension, sedentary activity and total cholesterol).^a^

		2003–2004(N = 1057)			2005–2006(N = 1082)			2007–2008(N = 1302)			2009–2010(N = 1370)	
Covariate	P value		OR(95% CI)	P value		OR(95% CI)	P value		OR(95% CI)	P value		OR(95% CI)
BPA	.251		1.03 (.978- 1.09)	.122		1.02 (.996- 1.04)	.867		.996 (.951- 1.04)	.163		1.00 (.998–1.01)
Age	**<.0001**		**1.08 (1.04- 1.12)**	**<.0001**		**1.11** **(1.07- 1.56)**	**<.0001**		**1.11** **(1.07–1.14)**	**<.0001**		**1.09** **(1.05–1.12)**
Gender	**.003**		**7.01** **(1.93- 25.4)**	**.0046**		**7.19** **(1.84- 28.2)**	**.005**		**3.75** **(1.49–9.44)**	**.008**		**3.70** **(1.39–9.80)**
BMI	.996		1.00 (.812- 1.23)	.073		1.18 (.985- 1.41)	**.040**		**1.15 (1.01–1.32)**	.924		.993 (.860–1.15)
Waist circumference	.842		.992 (.913- 1.08)	.245		.958 (.890–1.03)	.221		.964 (.910–1.02)	.428		1.02 (.966–1.09)
Family history: heart attack	**.0006**		**4.90** **(1.98- 12.2)**	.068		2.26 (.941–5.44)	.108		2.01 (.858–4.70)	**<.0001**		**4.72** **(2.24–9.90)**
Sedentary activity	.606		.939 (.739- 1.19)	.051		.775 (.599–1.00)						
Total cholesterol	**.045**		**.989 (.978- 1.00)**	**<.0001**		**.970 (.958-.983)**	**<.0001**		**.969 (.959-.979)**	**.002**		**.985 (.976-.994)**

a P values >0.05 for creatinine, ethnicity, income, hypertension for all four surveys. P values <0.05 for education and energy intake only for 2007–2008 survey; for smoking and heavy drinking only for 2005–2006 survey. Sedentary activity was excluded as a covariate for the 2007–2008 and 2009–2010 surveys as inclusion led to unacceptably small sample size.

**Table 3:** Association between BPA and heart attack using fully adjusted model (creatinine, age, gender, ethnicity, education, income, smoking, heavy drinking, BMI, waist circumference, energy intake, family history of heart attack, hypertension, sedentary activity and total cholesterol).^a^

	2003–2004(N = 1058)		2005–2006(N = 1083)		2007–2008(N = 1305)		2009–2010(N = 1373)	
**Covariate**	**P value**	**OR** **(95% CI)**	**P value**	**OR** **(95% CI)**	**P value**	**OR** **(95% CI)**	**P value**	**OR** **(95% CI)**
BPA	.073	1.04 (.996–1.09)	.107	1.02 (.996–1.04)	.602	.987 (.941–1.04)	.166	1.00 (.999–1.01)
Age	**.0002**	**1.07 (1.03–1.10)**	**.0254**	**1.04 (1.00–1.07)**	**<.0001**	**1.08 (1.05–1.11)**	**<.0001**	**1.06 (1.03–1.10)**
Gender	**.0002**	**10.9 (3.12–38.2)**	.811	.872 (.283–2.69)	.270	1.60 (.695–3.67)	.0784	2.27 (.911–5.64)
BMI	.953	.994 (.820–1.21)	.510	1.05 (.907–1.22)	.226	1.09 (.951–1.24)	.0620	.872 (.755–1.01)
Waist circumference	.547	1.02 (.949–1.10)	.990	1.00 (.940–1.06)	.469	.980 (.927–1.04)	**.0010**	**1.10 (1.04–1.17)**
Family history: heart attack	**.0009**	**4.79 (1.90–12.1)**	.0524	2.29 (.991–5.29)	**.0071**	**2.90 (1.34–6.29)**	**.0003**	**3.97 (1.88–8.40)**
Sedentary activity	.654	.949 (.755–1.19)	.507	.925 (.735–1.16)				
Total cholesterol	**.0468**	**.990 (.980–1.00)**	**<.0001**	**.975 (.964-.987)**	**<.0001**	**.977 (.968-.986)**	**.0194**	**.989 (.980-.998)**

a P values >0.05 for ethnicity, education, smoking, hypertension and creatinine for all four surveys and for energy intake for the three surveys with energy intake data. P values <0.05 for heavy drinking only for 2005–2006 survey and for income for 2005–2006, 2007–2008 and 2009–2010 surveys.

Sedentary activity was excluded as a covariate for the 2007–2008 and 2009–2010 surveys as inclusion led to unacceptably small sample size.

**Table 4:** Association between BPA and diabetes using fully adjusted model (creatinine, age, gender, ethnicity, education, income, smoking, heavy drinking, BMI, waist circumference, energy intake, family history of diabetes, hypertension, sedentary activity and total cholesterol).^a^

	2003–2004(N = 1039)		2005–2006(N = 1085)		2007–2008(N = 1316)		2009–2010(N = 1383)	
Covariate	P value	OR(95% CI)	P value	OR(95% CI)	P value	OR(95% CI)	P value	OR(95% CI)
BPA	.474	1.01 (.980–1.05)	.765	.993 (.950–1.04)	.173	.957 (.899–1.02)	.451	.993 (.975–1.01)
Age	**.006**	**1.03 (1.01–1.06)**	**<.0001**	**1.05 (1.03–1.08)**	**<.0001**	**1.05 (1.03–1.07)**	**<.0001**	**1.06 (1.04–1.08)**
Gender	.488	.785 (.397–1.55)	.664	.863 (.443–1.70)	.251	1.38 (.798–2.37)	.946	1.02 (.622–1.66)
BMI	**.020**	**.883 (.795-.980)**	.834	.990 (.905–1.08)	.475	1.03 (.949–1.12)	.496	1.03 (.949–1.11)
Waist circumference	**<.0001**	**1.11 (1.06–1.16)**	**.0154**	**1.05 (1.01–1.09)**	**.0372**	**1.04 (1.00–1.08)**	.110	1.03 (.994–1.06)
Family history: diabetes	**.0022**	**2.32 (1.35–3.95)**	**<.0001**	**3.30 (2.01–5.44)**	**<.0001**	**3.04 (1.97–4.67)**	**<.0001**	**2.94 (1.97–4.41)**
Hypertension	**.0012**	**2.68 (1.48–4.85)**	**.0044**	**2.14 (1.27–3.61)**	**.0003**	**2.21 (1.44–3.41)**	**<.0001**	**2.87 (1.87–4.44)**
Sedentary activity	.969	.997 (.854–1.16)	.528	1.05 (.906–1.21)				
Total cholesterol	.430	1.00 (.996–1.01)	.964	1.00 (.994–1.01)	**.0200**	**.994 (.989-.999)**	.144	.996 (.992–1.00)

a P values >0.05 for creatinine, smoking, heavy drinking for all four surveys and for energy intake for the three surveys with energy intake data. P values <0.05 for ethnicity and income only for 2005–2006 survey and for education for 2007–2008 only. Sedentary activity was excluded as a covariate for the 2007–2008 and 2009–2010 surveys as inclusion led to unacceptably small sample size.

Age and gender were statistically significantly associated with CHD in all four surveys (Table 2). The association with total cholesterol was statistically significant and inverse in all CHD analyses.

For heart attack (Table 3), the only factors showing consistent and statistically significant associations were age and total cholesterol. While frequency of heart attack increased with increasing age in all four surveys, total cholesterol was significantly inversely associated with heart attack for the four surveys (i.e., opposite of the expected direction).

In the analyses of diabetes (Table 4), adjusted ORs were statistically significantly increased for age, family history of diabetes, and hypertension in all four surveys.

When the data from four surveys were pooled, the ORs (95% CIs) for the full model that included all covariates were 1.004 (0.998–1.009) for CHD, 1.002 (0.998–1.007) for heart attack, and 0.995 (0.982–1.007) for diabetes. As shown in Table 5, the choice of covariates had only minor effect on point estimates. Although 95% CIs in some of the models did not cross unity there was no clear pattern to the results.

**Table 5:** Summary of odds ratios for pooled analyses using six alternative models with different sets of covariates.^a.^

Model	DiabetesOR (95% CI)	CHDOR (95% CI)	Heart attackOR (95% CI)
0	0.992 (0.979, 1.005)	1.004 (1.001, 1.008)	1.003(0.999, 1.007)
1	0.994 (0.981, 1.008)	1.005 (1.001, 1.009)	1.004 (1, 1.008)
2	0.994 (0.981, 1.008)	1.005 (1.001, 1.009)	1.004 (1, 1.008)
3	0.994 (0.979, 1.009 )	1.005 (1.001, 1.009)	1.004 (1, 1.008)
4	0.994 (0.979, 1.008)	1.004 (1, 1.009)	1.003 (0.999, 1.007)
5	0.995 (0.982, 1.007)	1.004 (0.998, 1.009)	1.002 (0.998, 1.007)

a Model 0 includes BPA and NHANES survey; Model 1 is Model 0 plus creatinine, age, gender, ethnicity, education and income; Model 2 is Model 1 plus smoking and drinking; Model 3 is Model 2 plus BMI and waist circumference; Model 4 is Model 3 plus hypertension and total cholesterol; and Model 5 is Model 4 plus family history.

### Comparison of Our Results and Methods with those of Previous Studies

A comparison of our methods and results to those of previous studies evaluating the relation between urinary BPA and diabetes, CHD and heart attack is presented in Table 6. Lang et al.’s [9] analysis of 2003–2004 NHANES data for diabetes gave an adjusted OR of 1.39 (95% 1.21–1.56), a result that is similar to the OR of 1.40 (95% CI, 1.25–1.60) reported by Melzer et al. [10], but different from ours (OR = 1.01; 95% CI, 0.98–1.05). Silver et al. [11] analyzed the same 2003–2004 survey using different diagnostic criteria and also reported a significantly increased OR of 1.23 (95% CI, 1.07–1.41). Melzer et al. [10] and Silver et al. [11] analyzed the 2005–2006 data for diagnosed diabetes and HbA1c/medication use, respectively, and found no evidence of an association with BPA, which is in agreement with the statistically non-significant OR of.993 in our study.

**Table 6:** Comparison of methods and results from this analysis and past research: BPA and diabetes, CHD, and heart attack (ORs for fully adjusted models).

Study	NHANES data	Exclusion criteria	Case definition	Covariates	OR, 95% CI, P value
**Diabetes**					
[9]	2003–04	Younger than 18, older than 74yrs. Missing urinary creatinine.	Combined physician-diagnosed diabetes and borderline diabetes	Age, gender, race/ethnicity, creatinine,education,income, smoking, BMI, WC	**1.39 (1.21–1.60), <.001**
[10]	2003–04	Younger than 18, older than 74yrs. Urinary BPA>80.1 ng/ml.Missing urinary creatinine.	Combined physician-diagnosed diabetes and borderline diabetes	Same as above	**1.40 (1.25–1.56),.00001**
	2005–06				1.02 (.76–1.38),.872
	pooled: 2003–04, 2005–06				**1.24 (1.10–1.40),.0001**
[11]	2003–04	Younger than 20 yrs. Urinary BPA>80.1 ng/ml. Those with missing important covariates.	HbA1c ≥6.5% or self-reporteduse of diabetes medication	Same as above	**1.23 (1.07, 1.41)**
	2005–06				1.06 (.95–1.19)
	2007–08				1.06 (.91–1.23)
	Pooled: 2003–04, 2005–06, 2007–08				**1.08 (1.02–1.16)**
Current study	2003–04	Those with missing covariates.	Physician-diagnosed diabetes; or fasting glucose >126 mg/dl or two-hour glucose tolerance test >200 mg/dl.	Same as above plus heavydrinking, fam. history ofdiabetes, hypertension,sedentary activity,cholesterol, energy intake(see Table 1)	1.01 (.980–1.05),.474
	2005–06				.993 (.950–1.04),.765
	2007–08				.957 (.899–1.02),.173
	2009–10				.993 (.975–1.01),.451
**Heart Attack**					
[9]	2003–04	Younger than 18, older than 74yrs. Missing urinary creatinine.	Physician-diagnosed heart attack	Age, gender, race/ethnicity, creatinine,education, income,smoking, BMI, WC	**1.40 (1.11–1.78),.008**
[10]	2003–04	Younger than 18, older than 74yrs. Urinary BPA>80.1 ng/ml.Missing urinary creatinine.	Physician-diagnosed heart attack	Same as above	**1.40 (1.07–1.84),.017**
	2005–06				1.39 (1.00–1.94).051
	pooled: 2003–04, 2005–06				**1.32 (1.15–1.52),.0003**
Current study	2003–04	Those with missing covariates.	Physician-diagnosed heart attack	Same as above plus heavydrinking, family history ofheart attack/angina,hypertension, sedentaryactivity, cholesterol,energy intake (see Table 1)	1.04 (.996–1.09),.073
	2005–06				1.02 (.996–1.04),.107
	2007–08				.987 (.941–1.04),.602
	2009–10				1.00 (.999–1.01),.166
**CHD**					
[9]	2003–04	Younger than 18, older than 74yrs. Missing urinary creatinine.	Physician-diagnosed CHD	Age, gender,race/ethnicity, creatinine,education, income,smoking, BMI, WC	**1.63 (1.18–2.26),.006**
[10]	2003–04	Younger than 18, older than 74yrs. Urinary BPA>80.1 ng/ml;Missing urinary creatinine.	Physician-diagnosed CHD	Same as above	**1.60 (1.11–2.32),.016**
	2005–06				**1.33 (1.01–1.75),.043**
	pooled: 2003–04, 2005–06				**1.42 (1.17–1.72).001**
Current study	2003–04	Those with missing covariates.	Physician-diagnosed CHD	Same as above plus heavy drinking, family history of heart attack/angina, hypertension, sedentary activity, cholesterol, energy intake (see Table 1)	1.03 (.978- 1.09),.251
	2005–06				1.02 (.996–1.04),.122
	2007–08				.996 (.951–1.04),.867
	2009–10				1.00 (.998–1.01),.163

For CHD and heart attack, Lang et al. [9] and Melzer et al. [10] observed almost identical associations in NHANES 2003–2004 data: 1.40 (95% CI, 1.11–1.78) and 1.40 (95% CI, 1.07–1.84) for heart attack, and 1.63 (95% CI, 1.18–2.26) and 1.60 (95% CI, 1.11–2.32) for CHD. The corresponding ORs (95% CIs) for heart attack and CHD in our study were 1.04 (0.996–1.09) and 1.03 (0.978–1.09), respectively. For the 2005–2006 survey data, Melzer et al. [10] reported that BPA was associated with heart attack (OR = 1.39; 95% CI: 1.00–1.94), although the result did not reach the conventional cutoff for statistical significance (p = .051), while the OR for CHD was significantly elevated (1.33; 95% CI, 1.01–1.75). The OR estimates for the 2005–2006 survey in our study were close to the null value (1.02 for both heart attack and CHD) and both 95% CIs included unity.

Table 6 also summarizes differences and similarities across the studies according to major methodological features: inclusion/exclusion criteria, outcome definition/ascertainment and inclusion of covariates. Unlike our study, which did not use any particular exclusions (except missing data), both Lang et al. [9] and Melzer et al. [10] excluded individuals under the age of 18 and over the age of 74 years, while Silver et al. [11] included participants ≥20 years of age. In addition, Melzer et al. [10] and Silver et al. [11] restricted their data to exclude participants with BPA levels >80.1 ng/ml. The studies also differed with respect to outcome definition for diabetes (Table 6), but the case definitions for CHD and heart attack in all studies, including ours, were the same. We controlled for additional covariates (heavy drinking, relevant family history, hypertension, sedentary activity, blood cholesterol, and daily energy intake) (Tables 1 and 6). One further difference relates to the reporting of ORs: Lang et al. and Melzer et al. gave ORs per 1 standard deviation change in BPA concentration, Silver et al. per doubling of BPA concentration, and in this research, per unit increase in BPA concentration.

## Discussion

In this paper, we used four NHANES datasets to assess inter-survey agreement with respect to associations between BPA and diabetes, CHD, and heart attack. We also compared our results with previous studies that addressed the same research questions based on the same NHANES surveys, but using slightly different methods of data selection, characterization and analysis. Finally, we used this research to address a larger question: Is NHANES an appropriate source of data for investigating the associations between chemicals with short physiologic half-lives such as BPA and chronic diseases with multi-factorial etiologies such as diabetes or heart disease?

### Methodological Issues in the Analyses of BPA and Health Outcomes using NHANES Data

In order to adequately compare our findings to those of Lang et al. [9] and Melzer et al. [10], we made sure we could reproduce their results. As described earlier, our primary goal was not to repeat past studies but to determine whether the use of a consistent scientifically and clinically supportable methodology yields coherent results across datasets. Nevertheless, our ability to reproduce previous results was important because it allowed us to assess the impact of different methodological and analytic decisions given the same data.

In assessing inter-survey agreement we expected, based on previous reports, that the 2003–2004 NHANES analyses would demonstrate a stronger association between BPA and outcomes of interest compared to more recent surveys. Our findings, however, were highly consistent across all four surveys, unexpectedly showing no associations for any of the outcomes.

Past analyses of the 2003–2004 and 2005–006 NHANES surveys produced different results from ours, leading to markedly different conclusions [9], [10]. The most plausible explanation for this discrepancy is differences in study methods. As discussed in the previous section, Lang et al. [9] and Melzer et al. [10] used more restrictive inclusion criteria (e.g., exclusion of participants older than 74 years and those at the high end of the urinary BPA distribution), defined diabetes as both physician-diagnosed and borderline diabetes, and included an appreciably shorter list of covariates.

An exploration of these methodological differences yielded important insights. First, we observed our adjustment for additional covariates that are known risk factors had no qualitative effect on the results (data not shown). This observation may be interpreted as either an indication that confounding by these covariates is not a source of bias in these data, or alternatively, evidence that information available in NHANES does not permit adequate control for confounders. Regardless of the interpretation, clearly inclusion of different covariates in the models did not explain the discrepancy between the two sets of results. We also found that the discrepancy between our findings on diabetes and those reported by Lang et al. [9] and Melzer et al. [10] was largely explained by the choice of case definition. Unlike our analyses, which compared persons with diabetes to all other subjects (including those with borderline diabetes), both earlier studies combined clinical diabetes and pre-diabetes into a single outcome category. Wei [36] raised concerns regarding this approach and proposed considering persons who met the criteria for the diagnosis of diabetes separately. In response, Melzer et al. [36] re-analyzed the 2003–2004 NHANES data excluding borderline diabetes and reported an attenuated OR of 1.19 (95% CI, 1.00–1.41; P = .05). Our analysis, which used the standard serum glucose levels for case definition of diabetes [18] and compared those who met the criteria for clinical disease to all other participants, found no significant association with urinary BPA. This is particularly important as those with borderline diabetes had the highest geometric mean urinary BPA concentration (compared to the other two groups) [36], thus indicating the lack of an expected dose-response relationship if BPA were truly associated with diabetes.

There were no differences in case definitions for CHD and heart attacks, yet our findings were in disagreement with those of Lang et al. [9] and Melzer et al. [10]. Comparing study methods, we found that this disagreement was attributable, in part, to differences in inclusion criteria. Melzer et al. [10] excluded persons with BPA levels above 80.1 ng/ml because “these high levels were outside the range of BPA in the original 2003/04 sample.” We know of no justification for excluding participants at the upper end of the urinary BPA distribution; therefore, we did not exclude individuals based on BPA levels. More importantly, we observed that the excluded individuals (N = 5) were all without CHD, and this exclusion biased the resulting OR away from the null; exclusion of disease-free individuals with the highest levels of exposure explains the observed disagreement between our results and those of Melzer et al.

### General Issues Related to the use of NHANES for Testing Causal Hypotheses

NHANES serves as an important source of data for determining the burden of chronic diseases and prevalence of risk factors in the US (http://www.cdc.gov/nchs/nhanes/about_nhanes.htm). According to CDC [37], NHANES biomonitoring data can be used “…so that appropriate studies can be conducted to determine whether these levels pose a health risk” (http://www.cdc.gov/exposurereport/faq.html). In this research, we showed how small, scientifically-supported changes in methodology can have critical consequences, resulting in inconsistent BPA-health outcomes associations. Rather than ascertaining which methodology and results are “superior,” these inconsistencies are used to highlight the larger question regarding whether use of NHANES data for studies of this type is appropriate, i.e., are observed associations, or lack thereof, meaningful?

The main limitation of cross-sectional studies such as NHANES is the inability to determine the temporal sequence of exposure and outcome, the main property of a cause-and-effect relation [38], [39]. While many NHANES-based studies include the caveat that the NHANES cross-sectional study design limits one’s ability to understand the true relationship between the exposure and the health outcome, the findings have often been interpreted as showing a link between various exposures and disease risk (rather than prevalence) thereby enabling causal interferences. Examples of implicit or explicit causal interpretations of NHANES data can be found in abundance in popular medical and science publications [40], [41] and the scientific literature [2]. Little attention appears to have been paid to a key issue raised by Goldberg and Silbergeld [42] for evaluating epidemiologic studies, namely whether a given study design and the available data are appropriate for the stated research question.

This issue is illustrated by our results pertaining to cholesterol levels. In all of our analyses, cholesterol levels were statistically significantly inversely associated with heart attack and CHD. Given the well-documented positive association between cholesterol and heart disease from prospective studies [43], [44], the most logical explanation for the observed result is reverse causation, i.e., it is likely that diagnoses of heart attack or CHD, which preceded the cholesterol measurements in NHANES, likely triggered changes in lifestyle or use of medications that resulted in lower cholesterol levels [45]. Exploration into the temporal aspect of the heart disease/cholesterol issue would require a different study design; the results from the cross-section design of NHANES give what appears to be a counter-intuitive finding.

This lack of temporal information impacts assessments of chemicals with short physiologic half-lives. BPA, with a half-life in the body of only a few hours, is just one of many short-lived chemicals that have been examined using NHANES databases for associations with chronic disease. Problems associated with the validity of NHANES BPA exposure measures for this type of evaluation were raised by Wolff [46] and apply to other short-lived chemicals as well: “Existing knowledge of exposure patterns as well as biomarker pharmacokinetics and consistency over time make it difficult to comprehend how concurrently measured BPA represents exposure across the latency period of a chronic disease.” Whether one-time measurements of chemicals with short physiologic half-lives can or should be used to ascertain chronic exposures must be carefully explored on a chemical-by-chemical basis [47]. However, it is clear that for many chemicals we cannot be confident that one-time measurements represent long-term exposures [48], [49].

### Conclusions

With scientifically and clinically supportable exclusion criteria and outcome definitions, we consistently found no associations between urinary BPA and heart disease or diabetes across four NHANES datasets. These results do not support associations and causal inferences reported in previous studies that used different criteria and definitions. To be clear, we are not drawing conclusions as to whether BPA is a risk factor for any of the chronic diseases discussed in this paper. In fact, we are stating the opposite - that using the NHANES surveys to draw such conclusions about short-lived environmental chemicals and chronic complex diseases is inappropriate. We need to expend resources on more appropriately designed epidemiologic studies and toxicological explorations to understand whether these types of chemicals play a causal role in chronic diseases.
