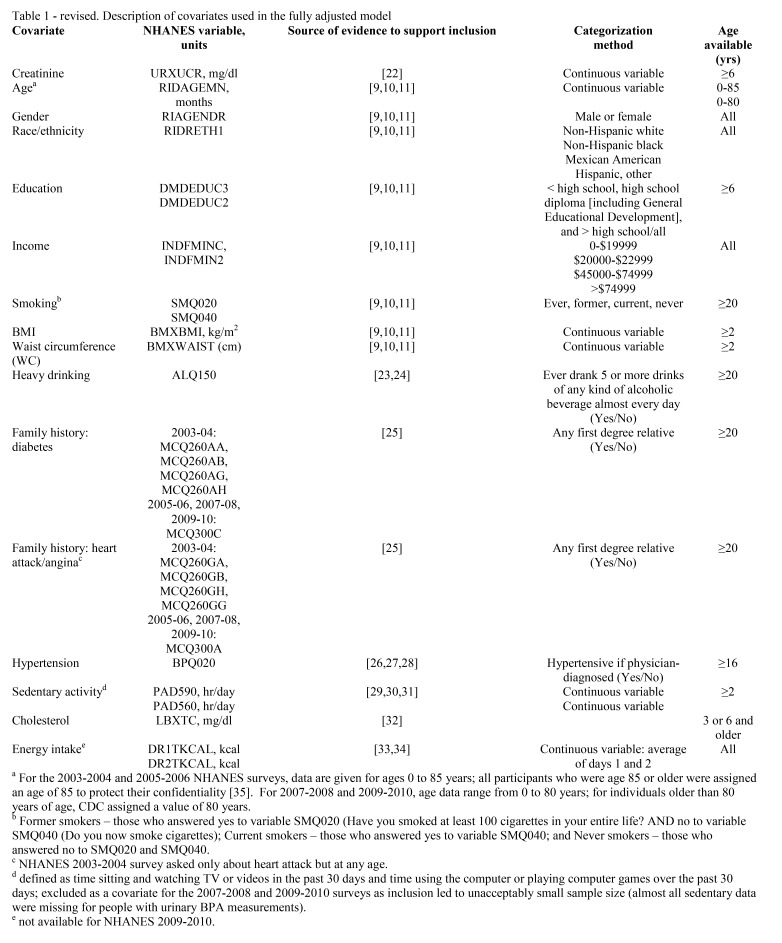# Correction: Use of NHANES Data to Link Chemical Exposures to Chronic Diseases: A Cautionary Tale

**DOI:** 10.1371/annotation/58af47b6-7a13-442d-b22b-86783ff12a4d

**Published:** 2013-05-03

**Authors:** Judy S. LaKind, Michael Goodman, Daniel Q. Naiman

There was an Error in Table 1. The correct Table can be found here: 

**Figure:**